# Positive association of *CD36* gene variants with the visual outcome of photodynamic therapy in polypoidal choroidal vasculopathy

**Published:** 2012-11-22

**Authors:** Shigeru Honda, Hiroaki Bessho, Naoshi Kondo, Sentaro Kusuhara, Yasutomo Tsukahara, Akira Negi

**Affiliations:** Department of Surgery, Division of Ophthalmology, Kobe University Graduate School of Medicine, Kobe, Japan

## Abstract

**Purpose:**

To clarify the association between *cluster of differentiation 36* (*CD36*) gene polymorphisms and the response to photodynamic therapy (PDT) in polypoidal choroidal vasculopathy (PCV).

**Methods:**

One hundred and thirty-seven patients with PCV were enrolled. The patients were treated with PDT and followed up for more than 6 months. Retreatments were performed every 3 months as needed based on findings from angiography. Patients who showed an improvement in their best-corrected visual acuity at 6 months post-PDT were classified as PDT responders, and the others were defined as non-responders. For the 73 responders and 64 non-responders, 19 single nucleotide polymorphisms (SNPs) across the *CD36* region were genotyped using the TaqMan assay. We analyzed the association between these variants and the visual outcomes of PDT.

**Results:**

The allelic frequencies of the SNPs rs3211851, rs3173798, and rs3211908 showed nominally significant differences between the PDT responders and non-responders. Genotype association analysis revealed a significant association of SNP rs3173798 with the visual outcome of PDT in a dominant model. The presence of the C allele in rs3173798 was significantly associated with a poor response to PDT after multivariate logistic regression analysis with clinical pre-PDT parameters. The mean best-corrected visual acuity in the group with the TT genotype of rs3173798 was significantly improved over 12 months of follow-up after the initial PDT.

**Conclusions:**

The coding variants in *CD36* are possibly associated with the visual outcome of PDT in patients with PCV.

## Introduction

Polypoidal choroidal vasculopathy (PCV) is a phenotype of age-related macular degeneration (AMD), accounting for 54.7% of patients with neovascular AMD in the Japanese population [1] and 24.5% in the Chinese population [2]. PCV has characteristic features such as orange-red protrusions at the posterior retinal and several distinct forms of choroidal vascular abnormalities, including vascular networks of choroidal origin with polypoidal lesions detected with indocyanine green angiography (ICGA) [3–5]. PCV often shows spontaneous regression during its natural course, but also often causes severe hemorrhagic and exudative changes that result in a poor visual prognosis [6].

PCV is known to have a better response to photodynamic therapy (PDT) with verteporfin than typical neovascular AMD, but the reason for this is not understood [7,8]. Moreover, there is some heterogeneity in the response to PDT among patients with PCV [9]. Recently, the genetic variants of rs10490924 (A69S) in the *age-related maculopathy susceptibility 2 (ARMS2)* gene and rs11200638 in the *high temperature requirement 1 (HTRA1)* gene have been reported to be associated with the effects of PDT in neovascular AMD and PCV [10–12]. However, we hypothesized that other candidate genes might be associated with the outcomes of PDT.

Cluster of differentiation 36 (CD36) is a multifunctional molecule that plays an important role in lipid metabolism, angiogenesis, inflammation, and scavenging oxidative stresses [13–15], all of which may be involved in the pathogenesis of AMD. A recent gene profiling study revealed that CD36 is expressed higher in the macula than in the peripheral retina [16]. In addition, the scavenging capability of CD36 for oxidative stress may be critical for the effects of PDT, since oxidative stress is widely recognized as an important component in the mechanism in which PDT works to occlude neovascular tracts [17]. We previously evaluated the association of coding variants in the *CD36* region with the incidence of typical neovascular AMD and PCV [18,19]. In those studies, variants of two single nucleotide polymorphisms (SNPs), rs3173798 and rs3211883, which are in high linkage disequilibrium, showed a significant association with typical neovascular AMD but not with PCV. However, since CD36 accelerates the uptake of oxidized low-density lipoprotein (oxLDL) [20], the verteporfin used in PDT binds with serum LDL, and this complex is incorporated into choroidal neovascularization (CNV) tissues [21], we hypothesized that the genetic variants in *CD36* may be associated with the effects of PDT. In this study, we genotyped 19 tag SNPs across the *CD36* region, and analyzed the association between these variants and the visual outcomes of PDT in a Japanese population.

## Methods

### Study participants

This study was approved by the Institutional Review Board at Kobe University Graduate School of Medicine, and was conducted in accordance with the Declaration of Helsinki. Written informed consent was obtained from all subjects. All cases in this study were Japanese individuals recruited from the Department of Ophthalmology at Kobe University Hospital in Japan.

The records of 137 patients with PCV treated with PDT and then followed up for more than 6 months were retrospectively reviewed. All of the patients consented to DNA sampling. All patients underwent ophthalmic examinations, including visual acuity measurements, slit-lamp biomicroscopy of the fundus, color fundus photography, optical coherence tomography (OCT), fluorescein angiography, and ICGA. All subjects with PCV enrolled in this study met the criteria for definite cases of PCV as proposed by the Japanese Study Group of Polypoidal Choroidal Vasculopathy [22]. Typically, subretinal reddish-orange protrusions corresponding to polypoidal lesions with/without vascular networks were detected with ICGA in the macular area. Patients who had received prior treatment for AMD were not included in this study.

The patients underwent standard PDT procedures as described previously [23]. The lesion status was assessed every 3 months, and treatments were performed again when serous retinal detachment, macular edema, or hemorrhage was recognized with funduscopy or OCT was accompanied by leakage on fluorescein angiography, or a defined lesion was observed on ICGA. If the patients with PCV showed improvement in their best-corrected visual acuity (BCVA) at 6 months post-PDT compared to baseline, they were classified as PDT responders. The other patients (who showed no improvement or a deterioration in their BCVA during the same period) were classified as PDT non-responders. Three patients who had complications (one patient with a severe increase in subretinal hemorrhage and two patients with retinal pigment epithelium [RPE] tears) after the initial PDT were included in the non-responder group since they showed no improvement in their BCVA at 6 months post-PDT. Patients with a baseline BCVA better than 20/25 or worse than 20/1000 were excluded from the analysis. Accordingly, 73 eyes from 73 responders and 64 eyes from 64 non-responders were subjected to further analysis. Every patient in this study received PDT in one eye during the study period. The details of the pretreatment factors of the PDT responders and non-responders are listed in Table 1.

**Table 1:** Clinical parameters of PDT responders and non-responders.

	Responder	Non-Responder	P value
Number of subjects	73	64	
Gender (male/female)	60/13	48/16	0.30 †
Age (years, mean±SD)	72.8±7.2	74.7±8.3	0.16 *
GLD (μm, mean±SD)	3730±1754	4133±1534	0.41 *
Baseline BCVA (logMAR)	0.70±0.37	0.60±0.32	0.10 *

GLD: greatest linear dimension, BCVA: best-corrected visual acuity, † χ2 test, * two-tailed unpaired *t* test.

### Single nucleotide polymorphism selection

Nineteen SNPs in the *CD36* region were selected based on our previous study [18], including rs3173798 and rs3211883, which were significantly associated with typical neovascular AMD but not with PCV. These SNPs were selected from the HapMap Project database [24] for the Japanese population using the tag selection tool [25] to capture 121 out of 123 SNPs with a minor allelic frequency above 0.1, with a mean r^2^ value of 0.97.

### Genotyping

Genomic DNA was extracted from the peripheral blood using the QIAamp DNA Blood Maxi Kit (Qiagen, Valencia, CA) and preserved at 4 °C until genotyping. Genotyping was performed using TaqMan SNP Genotyping Assays or Custom TaqMan SNP Genotyping Assays (Applied Biosystems, Foster City, CA) on a StepOnePlus Real-Time PCR System (Applied Biosystems) in accordance with the supplier’s recommendations. The final call rate across 137 samples over 19 SNPs genotyped was 100% in the present study.

### Statistical analysis

All SNPs were evaluated for Hardy–Weinberg equilibrium using the χ^2^ test (one degree of freedom) with SNPAlyze version 7.0.1 software (DYNACOM, Yokohama, Japan). The allelic and genotype frequency distributions were compared between the PDT responder and non-PDT responder subjects using a χ^2^ test with one or two degrees of freedom for the allelic and genotypic tests, respectively. Genotype association analyses were performed in the genotypic model, the dominant model (major allele homo versus hetero + minor allele mono), and the recessive model (major allele homo + hetero versus minor allele mono). Bonferroni corrections were applied for multiple testing. To exclude the influence of the clinical background before PDT, multivariate logistic regression analysis was performed with Stata 11 software (StataCorp LP, College Station, TX) including age, gender, pretreatment BCVA, and pretreatment greatest linear dimension, which may influence the outcomes of the PDT [8]. P values <0.05 were considered statistically significant.

## Results

None of the SNPs reported in the present study showed deviations from Hardy–Weinberg equilibrium over the entire sample (p>0.05). Table 2 summarizes the results from an allelic association study regarding the response to PDT. The nominally significant differences (with uncorrected p values) in the minor allelic frequencies were found at SNPs rs3211851, rs3173798, and rs3211908 between the PDT responders and non-responders. The statistical power of the allelic association analysis at each SNP was about 0.62, 0.70, and 0.57 (alpha error <0.05), for SNPs rs3211851, rs3173798, and rs3211908, respectively. In the genotype association analysis, the dominant models showed the nominally significant differences between PDT responders and non-responders for SNPs rs3211851 and rs3173798 (Table 3). However, only rs3173798 remained significant (p=0.011) after Bonferroni corrections over the 19 SNPs tested in this study. In contrast, the recessive models showed no significant differences between responders and non-responders. The statistical power of the genotype association analysis for a dominant model of rs3173798 was about 0.94 (alpha error <0.05). The results of the logistic regression analysis, which included the presence or absence of the C allele in the genotype of rs3173798 as an explanatory variable, conserved the statistical significance of this SNP (Table 4).

**Table 2:** Summary of the allelic association analysis regarding the response to PDT.

SNP ID	Location	Major/Minor allele	Minor allelic frequency (No. of genotypes: major homo/hetero/minor homo)		Association analysis results	
			Responder (n=73)	Non-responder (n=64)	Allele OR (95% CI)	P value
rs12531609	Intron 1	A/T	0.12 (56/16/1)	0.14 (48/14/2)	0.86 (0.43–1.72)	0.68
rs3211816	Intron 3	G/A	0.38 (28/35/10)	0.34 (27/30/7)	1.16 (0.70–1.92)	0.57
rs10499862	Intron 3	T/C	0.12 (56/16/1)	0.14 (48/14/2)	0.86 (0.43–1.72)	0.68
rs3211849	Intron 3	G/A	0.34 (35/26/12)	0.24 (36/25/3)	1.56 (0.94–2.61)	0.082
rs3211851	Intron 3	A/C	0.28 (39/27/7)	0.41 (20/35/9)	0.54 (0.32–0.92)	0.02
rs1054516	Intron 3	T/C	0.47 (22/33/18)	0.38 (25/29/10)	1.41 (0.88–2.26)	0.15
rs3173798	Intron 3	T/C	0.4 (29/29/15)	0.55 (9/39/16)	0.54 (0.33–0.88)	0.012
rs3211870	Intron 4	C/T	0.44 (21/40/12)	0.48 (16/34/14)	0.82 (0.49–1.35)	0.42
rs1358337	Intron 4	A/G	0.43 (24/35/14)	0.38 (23/34/7)	1.28 (0.78–2.11)	0.33
rs3211883	Intron 4	T/A	0.32 (37/26/10)	0.43 (19/35/10)	0.62 (0.38–1.01)	0.054
rs3173800	Intron 4	A/T	0.25 (41/27/5)	0.2 (40/23/1)	1.42 (0.79–2.57)	0.24
rs1924	Intron 5	G/A	0.22 (46/22/5)	0.3 (31/28/5)	0.67 (0.39–1.16)	0.15
rs17154232	Intron 6	G/C	0.12 (57/15/1)	0.12 (49/14/1)	0.92 (0.45–1.91)	0.83
rs17154233	Intron 6	A/C	0.16 (52/19/2)	0.11 (51/12/1)	1.51 (0.74–3.05)	0.25
rs3211908	Intron 7	C/T	0.14 (53/19/1)	0.24 (38/21/5)	0.54 (0.29–0.99)	0.042
rs17154258	Intron 8	A/G	0.15 (53/18/2)	0.2 (41/20/3)	0.70 (0.38–1.31)	0.26
rs1527483	Intron 11	G/A	0.2 (48/21/4)	0.3 (32/26/6)	0.60 (0.35–1.04)	0.068
rs3211958	Intron 14	A/G	0.37 (29/34/10)	0.42 (21/32/11)	0.80 (0.49–1.31)	0.38
rs7755	3′UTR	A/G	0.48 (22/32/19)	0.38 (21/38/5)	1.57 (0.95–2.60)	0.074

OR: Odds ratio, which represents the contribution of the minor allele to visual improvement; CI: coefficient interval.

**Table 3:** Summary of the genotype association analysis for selective SNPs associated with the response to PDT.

Genotype frequency (number of subjects)											
SNP ID	Major/Minor	Responder (n=73)			Non-responder (n=64)			Dominant model		Recessive model	
	allele	Major homo	Hetero	Minor homo	Major homo	Hetero	Minor homo	OR (95%CI)	P value (corrected P)	OR (95%CI)	P value (corrected P)
rs3211851	A/C	0.53 (39)	0.37 (27)	0.1 (7)	0.31 (20)	0.55 (35)	0.14 (9)	0.40 (0.20–0.80)	0.0085 (0.16)	0.65 (0.23–1.85)	0.42 (1.0)
rs3173798	T/C	0.4 (29)	0.4 (29)	0.2 (15)	0.14 (9)	0.61 (39)	0.25 (16)	0.25 (0.11–0.58)	6.0×10–4 (0.011)	0.78 (0.35–1.73)	0.53 (1.0)
rs3211908	C/T	0.73 (53)	0.26 (19)	0.01 (1)	0.59 (38)	0.33 (21)	0.08 (5)	0.55 (0.27–1.13)	0.1 (1.0)	0.16 (0.02–1.44)	0.057 (1.0)

OR: Odds ratio, which represents the contribution of the minor allele to visual improvement; CI: coefficient interval.

**Table 4:** Logistic regression analysis for the response to PDT including SNP rs3173798.

Explanatory variable	OR	95%CI	P value
Presence of C allele in genotype at rs3173798 (Yes=1, No=0)	0.28	0.12–0.67	0.0043
Age (years)	0.97	0.93–1.02	0.27
Gender (female=1, male=0)	0.92	0.39–2.20	0.85
Pre-treatment BCVA (logMAR)	1.49	0.50–4.43	0.47
GLD (μm)	1	1.00–1.00	0.88

BCVA: best-corrected visual acuity; GLD: greatest linear dimension; OR: odds ratio; CI: coefficient interval.

The clinical details of the patients with PCV stratified by their genotypes of rs3173798 in *CD36* are listed in Table 5. There was a significant difference in the male/female ratio among three genotype (TT, TC, and CC) groups. The age and mean number of PDT treatments per year tended to increase with the number of C alleles in the genotype of rs3173798, although there was no statistical significance.

**Table 5:** Clinical details of the PCV patients stratified by the genotype of rs3173798 in the CD36 gene.

	T/T (n=38)	T/C (n=68)	C/C (n=31)	P value
Gender (male/female)	35/3	53/15	20/11	0.020 †
Age (years, mean±SD)	72.7±7.9	73.3±7.7	75.8±7.6	0.21 *
GLD (μm, mean±SD)	3755±2058	4064±1631	3872±1145	0.78 *
Baseline BCVA (logMAR)	0.80±0.43	0.58±0.30	0.63±0.30	0.10 *
PDT frequency/year (mean±SD)	1.3±0.5	1.6±0.7	1.6±0.6	0.63 *

GLD: greatest linear dimension; BCVA: best-corrected visual acuity; PDT: photodynamic therapy. † χ2 test, * Kruskal–Wallis test.

**Figure 1:**
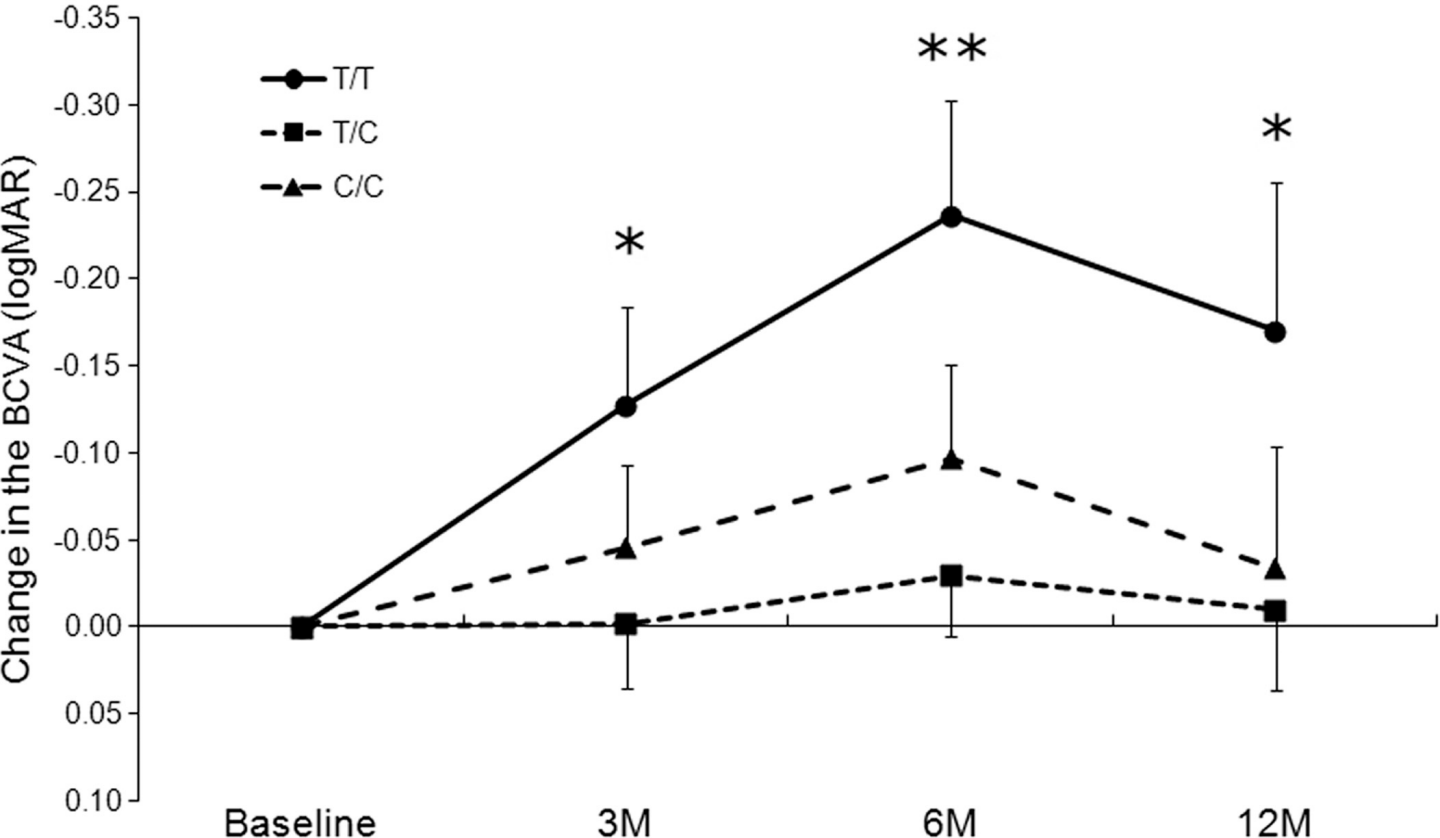
Influence of the genotype of rs3173798 in the *CD36* gene on the time-course of the logMAR best-corrected visual acuity value in patients with polypoidal choroidal vasculopathy treated with polypoidal choroidal vasculopathy. All values are presented as means±SEM. The time points in each genotype were compared against the baseline. * p<0.05, ** p<0.005.

In the time-course analysis, all patients (except three patients with a TC genotype) completed 12 months of follow-up after the initial PDT. The patients with PCV with a TT genotype showed significant improvements in their mean BCVA at 3, 6, and 12 months post-initial PDT (Figure 1). Patients with TC and CC genotypes showed no significant change in their mean BCVA up to 12 months post-initial PDT.

To assess the association of the genotype of rs3173798 with anatomic resolution of the lesion after PDT, additional analyses were performed using the following criterion to determine anatomic responders and non-responders. Patients with PCV who were successfully treated (complete resolution of the subretinal fluid and hemorrhage with complete or partial disappearance of the polypoidal lesions on angiography) with a single session of PDT were classified as anatomic responders. All others were classified as anatomic non-responders. In this analysis, the number of responders/non-responders was 14/17 with the CC genotype, 33/35 with the CT genotype, and 26/12 with the TT genotype at rs3173798. The difference between the CC+CT group and the TT group was statistically significant (p=0.035 using Fisher’s direct test).

## Discussion

We genotyped 19 SNPs across the *CD36* region in patients with PCV treated with PDT, and found that the genotype of rs3173798 in *CD36* was significantly associated with the patients’ visual outcome: patients with the TT genotype showed a significant improvement in vision after PDT. Although the results of the single SNP (allelic) association analysis revealed three candidate SNPs across the *CD36* region, the association of each SNP did not remain significant after the Bonferroni correction over the 19 SNPs tested in the present study. However, the results of the genotype association analysis demonstrated a significant association of rs3173798 with the visual outcome of PDT, even after the statistical corrections. These results may indicate a limitation for allelic association analysis in detecting possible genotype-phenotype associations in certain SNPs. Our previous studies demonstrated the association of SNPs rs3173798 and rs3211883 in the *CD36* region with a susceptibility to neovascular AMD [18,19]. In those studies, rs3173798 and rs3211883, which showed the most significant association with typical neovascular AMD, did not remain significant in the association with PCV. However, the present study suggested an independent association of the *CD36* variant with the visual outcome of PDT after logistic regression analysis with certain clinical pre-PDT factors. This may imply the existence of different pathophysiological roles for CD36 in the pathogenesis of PCV and in the mechanism by which PDT works, which suggests a complex association of this region with the phenotype of PCV, although the details have not been clarified yet. Interestingly, a recent report by Nakata et al. demonstrated an association of *pigment epithelium derived factor (PEDF)* gene variant rs12603825 with the effect of PDT in PCV [26]. However, they and other groups found no association of the SNPs in the *PEDF* gene (including rs1136287, which is in the same haplotype block as rs12603825) with the incidence of PCV [27–30]. These findings suggest that molecules that otherwise have no associations with the pathology of PCV may modulate the effects of PDT in patients with PCV.

The role of CD36 in PDT is currently unknown. Recent reports demonstrated that the coding variants in *ARMS2/HTRA1* can affect lesion size in neovascular AMD and PCV [31,32], which may influence the visual outcome post-PDT [8]. However, in the present study, the pretreatment greatest linear dimension was not different between the TT, TC, and CC genotypes at rs3173798. Moreover, the results of the logistic regression analysis suggested that the association of the coding variants at rs3173798 in *CD36* with the effects of PDT were independent of the clinical pretreatment factors evaluated. CD36 is involved in diverse physiologic and pathological processes, including scavenger receptor functions, transforming growth factor-β activation, lipid metabolism, angiogenesis, atherogenesis, and inflammation, depending on the ligands with which CD36 can interact [13–15]. In particular, the scavenging ability of CD36 against oxidative stress is critical for managing AMD, since oxidative stress is widely recognized as an important component in the pathogenesis of AMD [33,34] and in the mechanism by which PDT works to occlude neovascular tracts [17]. Recent in vitro studies have reported that CD36 is a key molecule in photoreceptor outer segment phagocytosis [35] and the uptake of oxLDL by RPE cells [20]. The incorporated oxLDL induces the expression of several genes related to oxidative stress, inflammation, and apoptosis in the RPE [36]. An immunohistochemical study has reported the presence of oxLDL in surgically excised CNV membranes [37]. Moreover, the verteporfin used in PDT binds with serum LDL, and this complex is then incorporated in the CNV tissues [38].

The biologic basis of the association between the genotype of rs3173798 and the function of CD36 is currently unknown, because the SNPs evaluated in the present study do not reside within the coding sequence of *CD36*. However, the FASTSNP program identified the sequence including rs3173798 as located in a potential splice site [39], and hence the SNPs in this region could have non-coding effects on gene expression and function. A recent study demonstrated that the C allele of rs3173798 tended to increase CD36 expression, which was correlated with an increase in low-density lipoprotein levels and a decrease in high-density lipoprotein levels in the serum [40,41]. Since the C allele of rs3173798 was more frequent in the non-responder group than in the responder group, the increased expression of CD36 might increase the uptake of verteporfin-bound LDL by RPE and thus cause PDT-induced damage in the RPE cells. Alternatively, the ability of CD36 to scavenge oxidative stress might attenuate the effects of PDT, which may be correlated with an insufficient anatomic resolution of the lesions in the CC and CT genotypes than in the TT genotype of rs3173798. However, a comprehensive reassessment of this locus may reveal potentially undiscovered and more important causative variants. In addition, it is essential to perform replication studies using other cohorts to verify the associations of the *CD36* variants with the effects of PDT. Moreover, there might be differences in the association between the *CD36* variants and PDT efficacy in other ethnic groups.

The limitation of this study was the small number of subjects enrolled and the relatively short follow-up period (1 year). Increasing the number of subjects is required to make a more robust conclusion about the association of the *CD36* gene with the outcome of PDT. Since evaluating the association of *CD36* variants with the durability of the PDT effect is important, further investigations with an extended follow-up period are needed.

Since PDT is known to induce several gene expression changes in the retina-choroidal complex [42], the detailed mechanisms by which multiple genes interact with each other to close the CNV are poorly understood. However, the present study suggested some clinical possibilities for genetic association analysis, which can be further investigated to determine the specific molecules involved in the mechanism(s) responsible for the actions of PDT, and may give genetic information that can be applied for personalized therapies in individual patients with PCV.
